# Congenital anomalies causing hemato/hydrocolpos: imaging findings, treatments, and outcomes

**DOI:** 10.1007/s11604-021-01115-7

**Published:** 2021-04-11

**Authors:** Keizo Tanitame, Nobuko Tanitame, Saiko Urayama, Kazuhiro Ohtsu

**Affiliations:** 1 Department of Diagnostic Radiology, Hiroshima Prefectural Hospital, Minami-ku, Ujinakanda, Hiroshima, 734-8530 Japan; 2 Department of Radiology, Hiroshima City Hiroshima Citizens Hospital, Hiroshima, Japan; 3 Department of Obstetrics and Gynecology, Hiroshima Prefectural Hospital, Hiroshima, Japan; 4 Department of Maternal and Child Health Research Center, Hiroshima Prefectural Hospital, Hiroshima, Japan

**Keywords:** Hematocolpos, Imperforate hymen, Distal vaginal agenesis, Transverse vaginal septum, OHVIRA

## Abstract

Hemato/hydrocolpos due to congenital urogenital anomalies are rare conditions discovered in neonatal, infant, and adolescent girls. Diagnosis is often missed or delayed owing to its rare incidence and nonspecific symptoms. If early correct diagnosis and treatment cannot be performed, late complications such as tubal adhesion, pelvic endometriosis, and infertility may develop. Congenital urogenital anomalies causing hemato/hydrocolpos are mainly of four types: imperforate hymen, distal vaginal agenesis, transverse vaginal septum, and obstructed hemivagina and ipsilateral renal anomaly, and clinicians should have adequate knowledge about these anomalies. This article aimed to review the diagnosis and treatment of these urogenital anomalies by describing embryology, clinical presentation, imaging findings, surgical management, and postoperative outcomes.

## Introduction

**Fig. 1:**
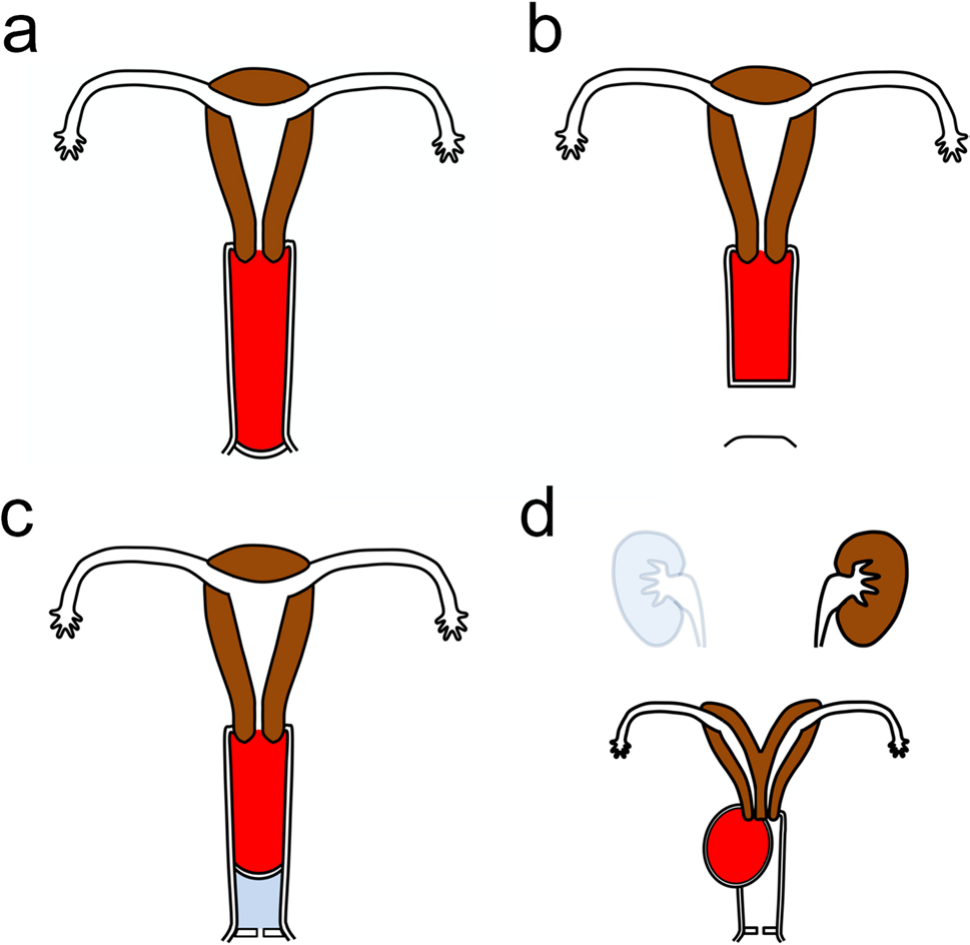
Schematic illustrations show coronal views of congenital urogenital anomalies causing hematocolpos. The accumulated blood in the vagina is colored in red. **a** Imperforate hymen. **b** Distal vaginal agenesis. **c** Complete transverse vaginal septum. **d** Obstructed hemivagina and ipsilateral renal anomaly (OHVIRA)

Hemato/hydrocolpos is a medical condition in which menstrual blood or secretory fluid accumulates in the vagina due to vaginal obstruction. Hemato/hydrocolpos are caused by congenital urogenital anomalies or acquired vaginal occlusion due to infection, trauma, or sexual abuse [1]. There are mainly four congenital causes: imperforate hymen, distal vaginal agenesis, complete transverse vaginal septum, and obstructed hemivagina and ipsilateral renal anomaly (OHVIRA), the schemas of which are shown in Fig. 1.

Hydrocolpos due to congenital urogenital anomalies could be discovered during the prenatal and postnatal periods owing to the collection of secretions in the obstructed vagina under the influence of maternal hormones [2, 3]. However, most patients are detected at puberty with several symptoms due to hematocolpos or hematometrocolpos [4–7]. Clinical symptoms are often nonspecific and include suprapelvic tender mass, cyclical lower abdominal pain, constipation, vomiting, and urinary retention [8–10]. Thus, such patients could be misdiagnosed as acute appendicitis, ovarian torsion, or urinary infection [11, 12].

If early diagnosis and treatment are achieved, the long-term outcomes are generally good. In contrast, without early and proper treatment, late complications such as tubal infection, adhesion, pelvic endometriosis, infertility, and renal failure secondary to hydronephrosis can develop [13–15].

Ultrasonography, computed tomography (CT) and magnetic resonance imaging (MRI) are used to evaluate patients with hemato/hydrocolpos. In particular, MRI, with its high-resolution and soft tissue contrast, provides useful information for the differential diagnosis of obstructive causes and management decisions [16, 17].

Surgical treatment is necessary for patients with hemato/hydrocolpos due to congenital vaginal obstruction. Although drainage of accumulated blood or secretions in the vagina should be carried out, long-term transvaginal drainage may induce the risk of retrograde infection. The uterus should not be squeezed for drainage as tubal adhesions and peritoneal endometriosis due to endometrial cell dissemination may lead to infertility [18, 19].

We herein review and discuss the embryology, clinical and imaging findings, treatment options, and outcomes of previously described congenital anomalies causing hemato/hydrocolpos, which is helpful to make early diagnosis and treatment possible.

### The embryology of vaginal development

The upper parts of the Müllerian ducts are unfused and form fallopian tubes. In contrast, the lower parts of the Müllerian ducts fuse and finally form the uterus and upper part of the vagina.

**Fig. 2:**
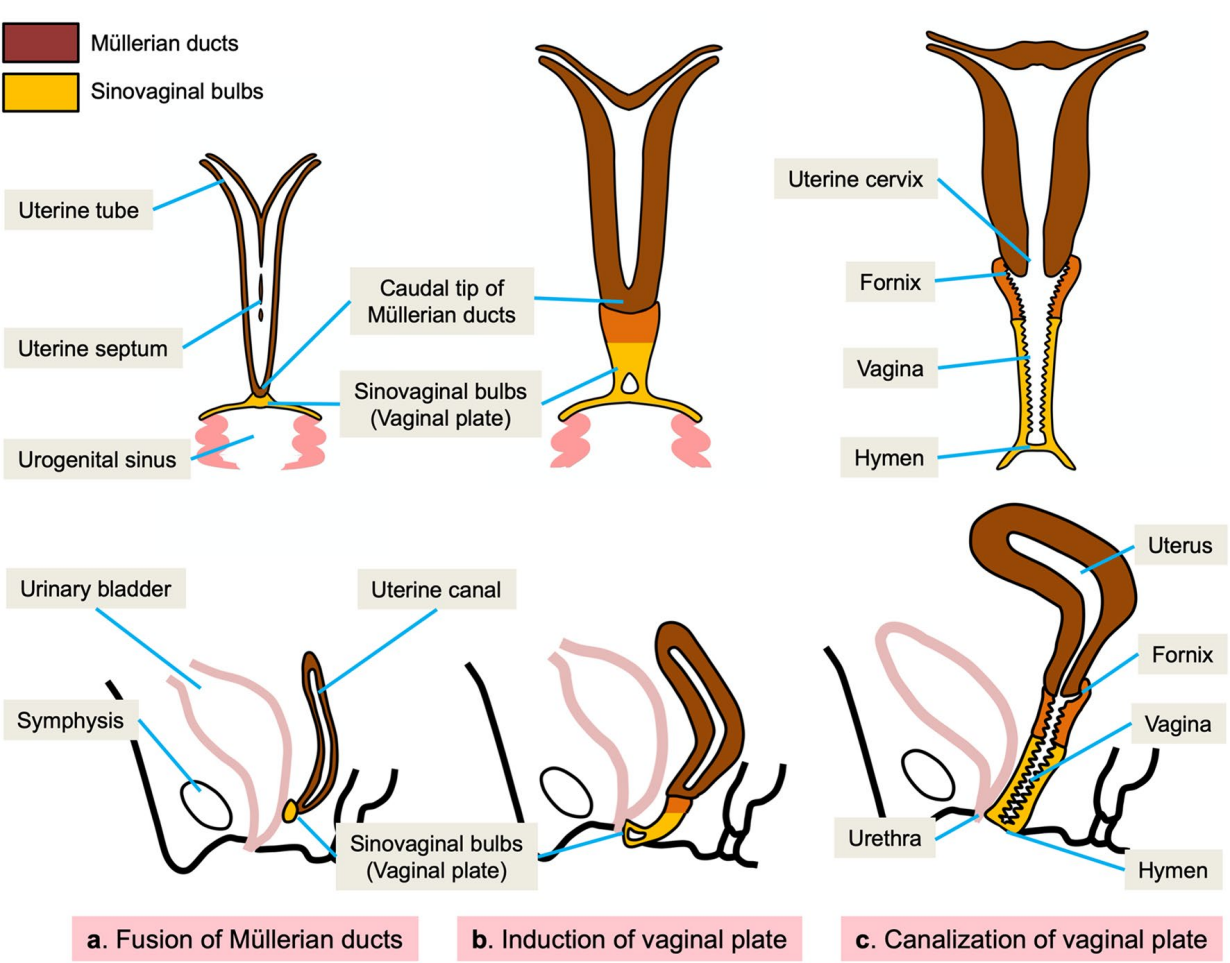
Development of the vagina. **a** After the caudal tip of the fused Müllerian ducts reaches the urogenital sinus, a sinovaginal bulb grows out of the sinus. **b** The sinovaginal bulb proliferates and forms a solid vaginal plate. Proliferation continues at the cranial end of the plate. **c** By the 5th month, the vaginal plate is entirely canalized and forms the vagina

The uterovaginal development is shown in Fig. 2 [20]. After the caudal tip of the fused Müllerian ducts reaches the urogenital sinus, sinus-derived endodermal cells proliferate and form a solid sinovaginal bulb. The sinovaginal bulb proliferates and forms a vaginal plate. Proliferation continues at the cranial end of the plate and increases the distance between the developing uterus and urogenital sinus, and the core of the sinovaginal bulb degenerates and forms a cavity at 17–18 week’s gestation. By the 5th month of pregnancy, the vaginal plate is completely canalized. The upper and lower parts of the vagina are considered to be derived from the Müllerian ducts and sinovaginal bulbs, respectively.

The hymen is a thin mucosal tissue between the urogenital sinus and the sinovaginal bulb. A small opening is generally developed in the hymen during perinatal life [19].

### Congenital anomalies

#### Imperforate hymen

In imperforate hymen, the vaginal orifice is occluded by a hymen without an opening. The prevalence of imperforate hymen is estimated at one in 1000 to one in 2000 females [5, 21]. Although rare cases of familial imperforate hymen have been reported [11, 22], most cases are thought to occur sporadically.

In female neonates with an imperforate hymen, hydrocolpos or hydrometrocolpos caused by maternal estrogen are incidentally discovered [23, 24]. However, most of the patients with imperforate hymen present with primary amenorrhea, cyclical pelvic pain or urinary retention secondary to hematocolpos or hematometrocolpos at puberty [8, 19, 25, 26].

**Fig. 3:**
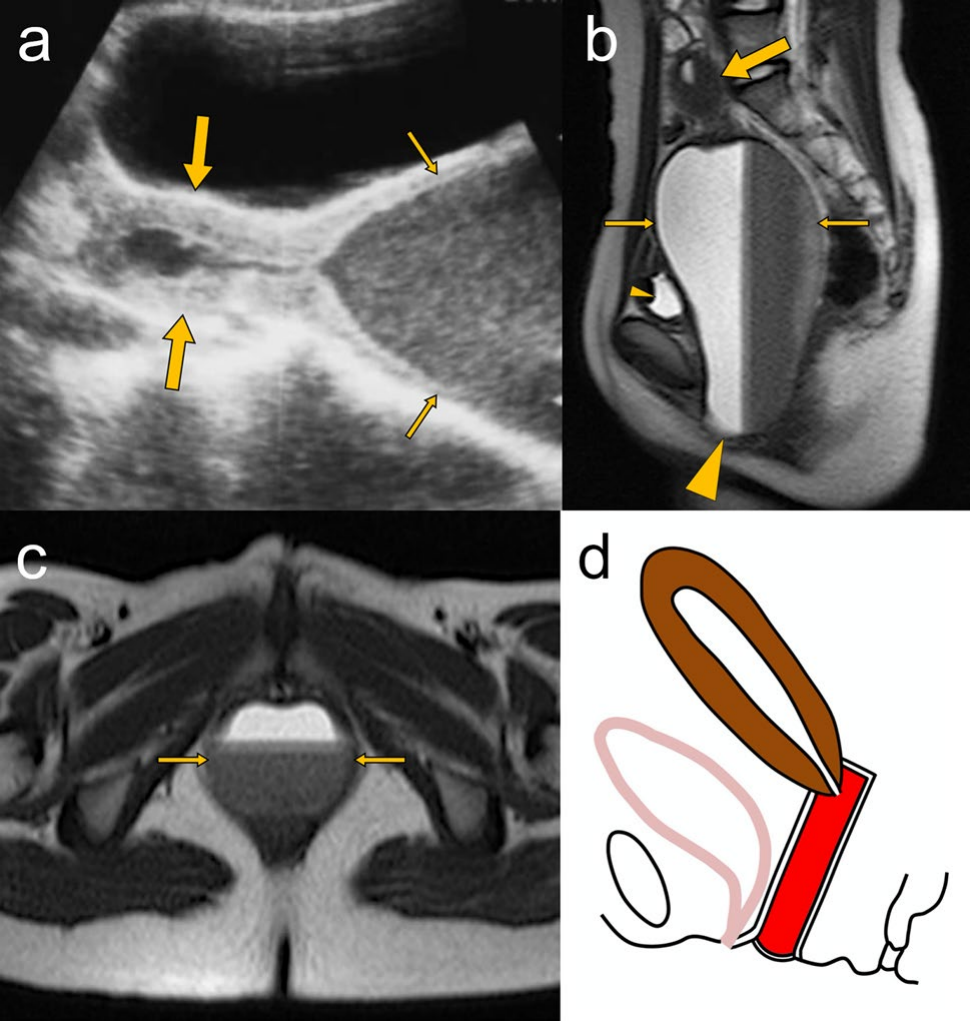
A 13-year-old girl with imperforate hymen. **a** Abdominal ultrasonography shows a normal-shaped uterus (large arrows) and a distended vagina (small arrows) containing echogenic fluid. **b** Sagittal T2-weighted MRI image shows the distended vagina (small arrows) measuring 14.5 × 7.5 cm in size with a fluid–fluid level (“hematocrit effect”), which compresses the bladder (small arrowhead). The bulging imperforate hymen (large arrowhead) protrudes between the labia. The uterus (large arrow) is normally visualized. **c** Axial T2-weighted MRI image shows the distended vagina (small arrows) containing blood products between the urethra and anal canal. **d** Schematic illustration shows a sagittal view of hematocolpos due to imperforate hymen. Intravaginal blood is colored in red

Hematocolpos due to imperforate hymen can be diagnosed easily by perineal inspection, which reveals bulging, bluish hymen without a vaginal opening [19, 27, 28]. However, this condition can be easily missed if a careful recording of history and perineal examination are not performed. Abdominal ultrasonography can reveal hemato/hydrocolpos (Fig. 3a), and MRI can depict the whole vagina distended with hematoma and the bulging hymen protruding from the introitus (Fig. 3b, c). MRI is a valuable imaging tool for assessing the extent of hematocolpos, hematometra and/or hematosalpinx, the thickness of the imperforate hymen, and related complications such as infection, hydronephrosis and endometriosis [5, 29]. The schematic illustration of the imperforate hymen is shown in Fig. 3d.

Imperforate hymen is generally managed by hymenotomy (surgical incision of the hymen) or hymenectomy (surgical removal of the hymen). Although no differences in outcomes between the two surgical methods exist, Lee et al. [11] reported that hymenectomy was associated with more frequent complications than hymenotomy. Hymen-preserving surgeries such as simple vertical incision and annular hymenotomy are occasionally performed owing to the importance of the first intercourse bleeding [27, 30]. Although complications such as re-closure, vaginal adenosis, or vaginal adhesion are reported to occur in only 6.6% of postsurgical patients [11], careful follow-up is necessary to ensure no recurrence and complications. Long-term outcomes after adequate surgery for imperforate hymen are good [11, 21, 27].

Prepubertal diagnosis of imperforate hymen provides some benefits such as optimal timing of surgery and avoidance of severe complications related to delayed treatments namely, tubal adhesions, pelvic endometriosis, and infertility. Considering that the diagnosis of imperforate hymen is easily made by inspection of female genitalia, pediatricians should incorporate the genital inspection of prepubertal females in routine clinical practice [31].

#### Distal vaginal agenesis

The prevalence of vaginal agenesis has been reported to be one in 4000 to one in 10,000 females [32]. Vaginal agenesis is classified as complete, proximal, and distal agenesis [33], with the latter, estimated to be 5% of the total number. As the lower segment of the vagina develops from the urogenital sinus, distal vaginal agenesis is the failure of the urogenital sinus to form the lower segment of the vagina or partial failure of the vaginal plate to canalize. According to the American Society for Reproductive Medicine (ASRM) classification, distal vaginal agenesis is classified as a class IA anomaly [34].

Most patients with distal vaginal agenesis present with several symptoms due to the retention of menstrual blood at puberty. Distal vaginal agenesis, as well as imperforate hymen, can be diagnosed during genital inspection; ideally, this condition should be recognized during neonatal and prepubertal periods to determine the optimal timing of surgery and avoid complications related to delayed treatment [6].

**Fig. 4:**
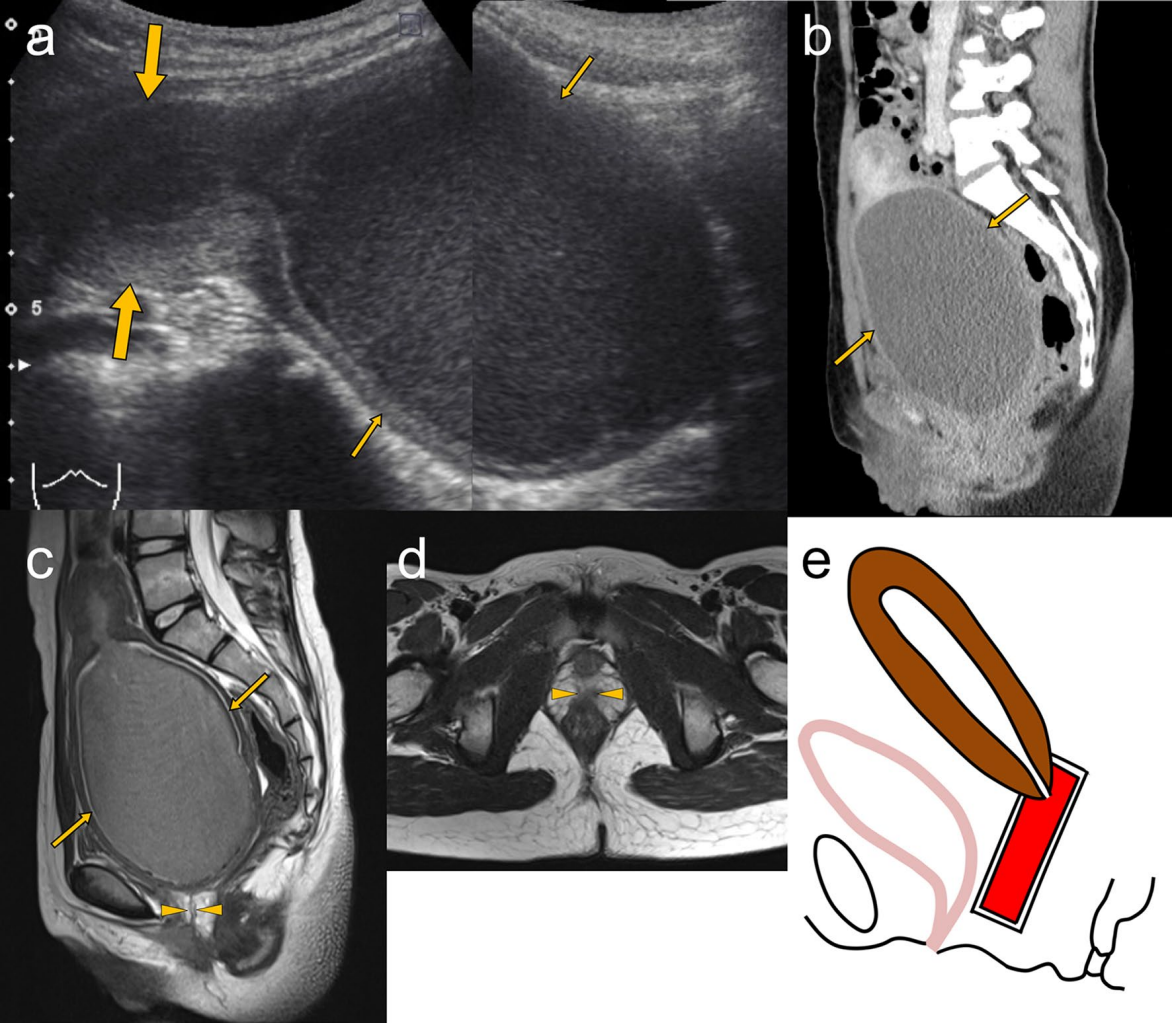
An 11-year-old girl with lower vaginal agenesis. **a** Abdominal ultrasonography shows a normal-shaped uterus (large arrows) and a grossly distended upper vaginal part (small arrows) with moving internal echoes. **b** Sagittal reformatted contrast-enhanced CT image shows the distended upper part of the vagina (small arrows) containing slightly hyperdense fluid. **c**, **d** Sagittal (**c**) and axial (**d**) T2-weighted MRI images show the distended upper vagina (small arrows) containing blood products and absence of the lower vagina with replacement by small fibrous tissue (arrowheads). MRI is better than CT in demonstrating distal vaginal agenesis. **e** Schematic illustration shows a sagittal view of hematocolpos due to distal vaginal agenesis. The accumulated blood in the upper part of the vagina is colored in red

Perineal inspection reveals the absence of hymen and vaginal orifice; however, with a small concave dimple [6, 9, 35]. In patients with hematocolpos, transabdominal ultrasonography can reveal the distended upper part of the vagina with moving internal echoes (Fig. 4a), and ultrasonography with a transperineal approach may demonstrate the length of the atretic vaginal segment [6, 36]. Although distal vaginal agenesis can also be suggested using a CT scan (Fig. 4b), MRI can better delineate the atretic vaginal segment (Fig. 4c, d), which allows the differential diagnosis from the more common hematocolpos due to the imperforate hymen. MRI is essential for the morphological assessment and surgical planning of distal vaginal agenesis (tailoring the length of the graft) [9, 35, 37]. The schematic illustration of distal vaginal agenesis is shown in Fig. 4e.

In surgical treatment, local pull-through vaginoplasty with direct anastomosis of the upper vaginal mucosa to the introitus via a perineal incision is performed when the distance between the perineal surface and the caudal aspect of the distended vagina is 2 cm or less [6, 9, 37–39]. Vaginoplasty using skin or bowel grafts has been performed when a large segment of the vagina is absent [6, 9, 35–37]. Fibrin glue improves graft stability [6, 40].

The timing of surgery is controversial. If the diagnosis is made during the neonatal or prepubertal periods, curative surgery should be postponed until hematocolpos develop at menarche. This is owing to larger dilated upper vaginal segments making it easier to determine the best surgical incision route and decrease the graft length [37].

**Fig. 5:**
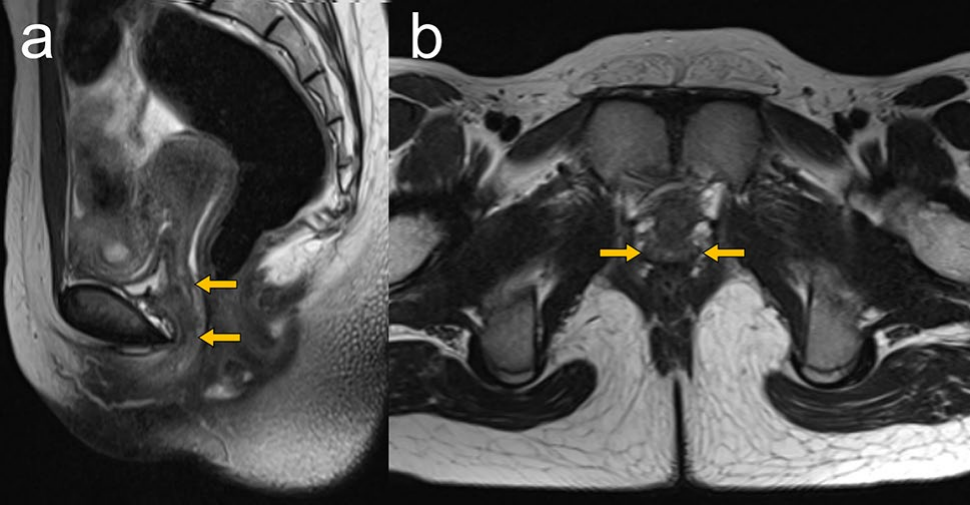
An 11-year-old girl after vaginoplasty for distal vaginal agenesis. Sagittal (**a**) and axial (**b**) T2-weighted MRI images show a neovagina (arrows). No postoperative complications, such as hydrometra due to vaginal stenosis or vaginal shortening, are observed

Postoperative vaginal stricture after a vaginoplasty is the most common late complication and adversely affects the patient’s sex life and pregnancy. Vaginal prosthesis, inflatable silicone stents, and estrogen ointment are used to avoid vaginal stricture after surgery [6, 9]. MRI is a useful imaging modality to exclude postoperative complications such as hydrometrocolpos due to vaginal stenosis or vaginal shortening (Fig. 5).

#### Transverse vaginal septum

An incomplete vaginal septum is infrequently observed, and a complete septum is quite rare. The prevalence of transverse vaginal septum is reported to be one in 30,000 to one in 84,000 females [41]. Although it can be located at any level of the vagina, the most common location of the transverse septum is the upper vagina (46%), followed by the mid vagina (35%) and the lower vagina (14%) [42]. A transverse vaginal septum, a class IA anomaly according to the ASRM classification, is assumed to occur due to the failure of tissue resorption between the sinovaginal bulb and the caudal tip of the fused Müllerian ducts [43]. This septum is a membrane of fibrous connective tissue with vascular and muscular components [41]. Thicker septa are more commonly found closer to the uterine cervix [13].

In patients with upper or mid vaginal septum, no abnormalities are observed during genital examination. A transverse vaginal septum is generally diagnosed in the affected individuals presenting with primary amenorrhea and cyclical pelvic pain due to hematocolpos. Although early diagnosis and intervention of the transverse vaginal septum can lead to a better outcome, early and accurate diagnosis before menarcheal age is still challenging [13].

**Fig. 6:**
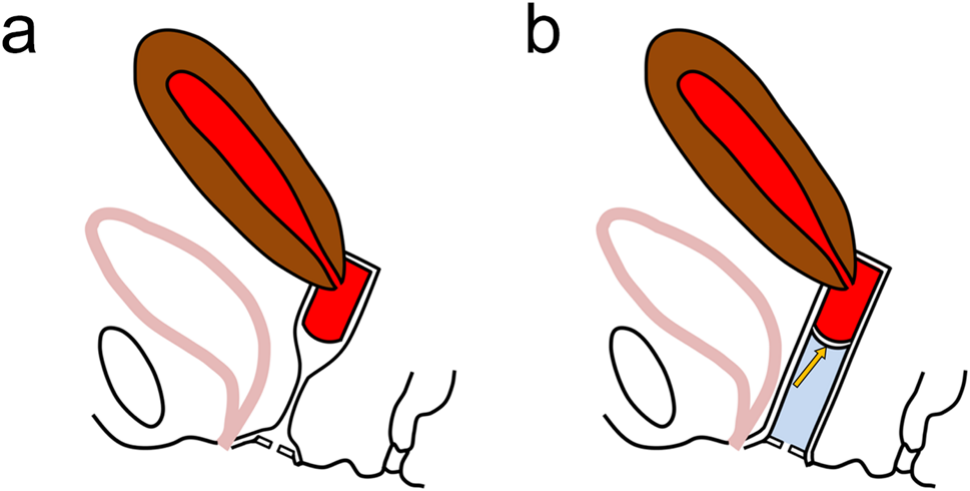
Instilling of jelly through the vaginal introitus for evaluating transverse vaginal septum. **a** Schematic illustration shows a sagittal view of hematometrocolpos due to transverse vaginal septum. There is a difficulty to distinguish the vaginal septum from the collapsed lower vagina. **b** After instilling of jelly through the vaginal introitus, hematometrocolpos (colored in red), the vaginal septum (arrow), and the lower vaginal segment containing jelly (colored in blue) are clearly shown. Intravaginal infusion of jelly has been reported to provide information on the level and thickness of the vaginal septum

In patients with hemato/hydrocolpos, abdominal ultrasonography can reveal the distended upper part of the vagina. MRI can depict the distended upper part and the collapsed lower part of the vagina in patients with a transverse vaginal septum, which allows the differential diagnosis from hematocolpos due to imperforate hymen or vaginal agenesis [13]. However, it is difficult to distinguish the vaginal septum from the collapsed normal lower vaginal wall using conventional MRI. Infusion of an adequate volume of ultrasound jelly through the vaginal introitus can expand the lower segment of the vagina and lead to better MRI delineation of the vaginal septum [10, 35, 43]. This technique has been reported to be useful for measuring the level and thickness of the vaginal septum. The schematic illustration is shown in Fig. 6.

Vaginal septa should be completely removed before end-to-end anastomosis of the upper and lower vagina. Double-crossed Z-plasty using eight vaginal mucosal flaps is frequently performed to decrease the risk of postoperative stenosis [44–46]. When end-to-end anastomosis is difficult due to thick vaginal septa, a biological graft is required to reconstruct a normal-sized vagina [13, 35, 45, 46]. A vaginal mold or silicone stent is often placed in the postoperative vagina to maintain patency [13, 43]. Postoperative vaginal stenosis is the most common complication. However, successful surgery can lead to satisfactory sex life in patients.

### OHVIRA

The prevalence of OHVIRA is still unknown; however, it is estimated to be one in 4000 to one in 50,000 females [47]. OHVIRA is a congenital anomaly associated with the abnormal development of the Müllerian and Wolffian ducts, which is characterized by the triad of didelphys uterus, obstructed hemivagina and ipsilateral renal agenesis. Although OHVIRA may be known as Herlyn–Werner–Wunderlich syndrome (HWWS), HWWS has some variants, and Wolffian duct anomalies in HWWS include renal agenesis, duplicated kidneys, dysplastic kidneys, crossed fused renal ectopia, and rectovesical bands.

Right-sided dominance of OHVIRA has also been reported [47–50]. According to the ASRM classification, OHVIRA is considered a combined class IA vaginal anomaly and class III uterine anomaly with unilateral renal agenesis. Zhu et al. [51] described that OHVIRA can be classified into two types according to complete or incomplete obstruction of the hemivagina.

OHVIRA with complete vaginal obstruction is generally discovered at puberty with symptoms such as cyclical pelvic pain and dysmenorrhea due to retention of menstrual blood. In contrast, in patients with incomplete vaginal obstruction, regular menstruation and slow extension of hematocolpos lead to delayed diagnosis [52, 53], and pyometrocolpos might occur due to secondary infection of accumulated fluid. Infection in the incompletely obstructed hemivagina often flares during pregnancy, probably owing to increased glandular activity [54].

**Fig. 7:**
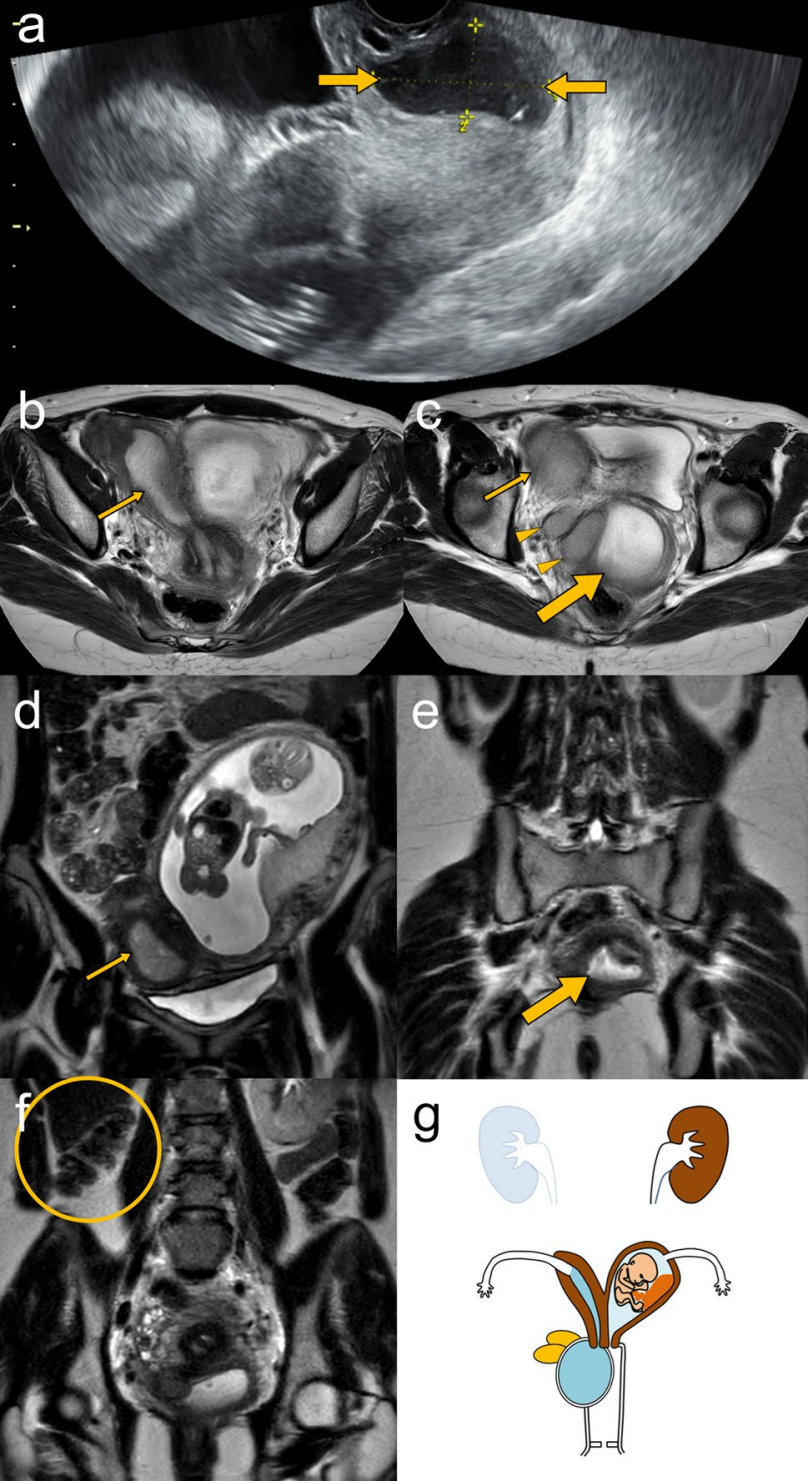
A 30-year-old pregnant woman at 18 weeks of gestation having right obstructed hemivagina and ipsilateral renal anomaly (OHVIRA) with pyometrocolpos. **a** Transvaginal ultrasonography shows a distended right hemivagina containing hypoechoic fluid (arrows). **b**–**f** Axial (**b**, **c**) and coronal (**d**, **e**) T2-weighted MRI images show duplication of the uterine and vaginal canals. The distended right hemivagina (large arrows), right uterine horn (small arrows), vaginal retention cysts (arrowheads), and a fetus at 17 weeks in the left uterine horn are identified. Coronal T2-weighted MRI image (**f**) also demonstrates the absence of the right kidney (oval circle). **g** Schematic illustration shows a coronal view of the right-sided OHVIRA with pregnancy in the left uterus

The inspection of external genitalia is generally unremarkable. Abdominal and transvaginal ultrasonography can depict ipsilateral renal agenesis, uterovaginal duplication, and distended hemivagina (Fig. 7a). MRI with multiplanar image acquisition provides detailed information of OHVIRA syndromes such as the external uterine contour, the location and thickness of the obstructed hemivaginal septum, and associated abnormalities such as endometriosis, pelvic inflammation and adhesions (Fig. 7b–f) [3, 47, 49, 53, 55]. Vaginal speculum examination could be useful for differentiating between complete and partial obstruction of hemivagina. The schema of OHVIRA with pyometrocolpos in a pregnant woman is shown in Fig. 7g.

Resection of the vaginal septum to relieve obstruction is the treatment of choice, and full resection of the vaginal septum is considered to achieve good outcomes and fertility [51]. Postoperative complications are uncommon; however, vaginal adenosis, vaginal stenosis, and re-closure of the vaginal septum have been reported [53, 55].

Hemihysterectomy of an obstructed uterine horn is not recommended owing to the possibility of fertility in a previously obstructed uterus [56]. Women with uterus didelphys have a high pregnancy rate of 80%; however, elevated rates of premature delivery (22%), miscarriage (74%), and cesarean section (over 80%) are observed [47, 54, 57].

Early diagnosis and surgical treatment can prevent complications and preserve future fertility. The anomalous differentiation of Wolffian and Müllerian ducts can cause renal abnormalities, and the most common presentation is renal agenesis. The prevalence of unilateral renal agenesis is 1/1100, and over 30% of affected women exhibit associated ipsilateral Müllerian anomalies [47, 58]. If unilateral renal agenesis or other renal abnormalities are found in newborn, infant and prepubertal females, OHVIRA and other Müllerian anomalies frequently accompanied with the condition should be suspected [47, 54, 57].

## Conclusions

In this review, we described the embryology, imaging findings and treatment options for congenital urogenital anomalies causing hemato/hydrocolpos namely, imperforate hymen, distal vaginal agenesis, complete transverse vaginal septum, and OHVIRA. Early correct diagnosis and treatment reduces the risk of pelvic endometriosis. Therefore, radiologists should be familiar with these imaging features, and other clinicians, particularly pediatricians, gynecologists, and urologists, should consider the possibility of these urogenital anomalies in clinical practices for newborn, infant and prepubertal females.
